# Liquiritigenin Reduces Blood Glucose Level and Bone Adverse Effects in Hyperglycemic Adult Zebrafish

**DOI:** 10.3390/nu11051042

**Published:** 2019-05-09

**Authors:** Marta Carnovali, Livio Luzi, Ileana Terruzzi, Giuseppe Banfi, Massimo Mariotti

**Affiliations:** 1 Gruppo Ospedaliero San Donato Foundation, 20122 Milan, Italy; marta.carnovali@grupposandonato.it; 2 Policlinico San Donato IRCCS, 20097 Milan, Italy; livio.luzi@unimi.it; 3 Department of Biomedical Sciences for Health, University of Milan, 20133 Milan, Italy; 4 Diabetes Research Institute, Metabolism, Nutrigenomics and Cellular Differentiation Unit, San Raffaele Scientific Institute, 20132 Milan, Italy; terruzzi.ileana@hsr.it; 5 IRCCS Orthopedic Institute Galeazzi, 20161 Milan, Italy; banfi.giuseppe@fondazionesanraffaele.it; 6 Faculty of Medicine and Surgery, Vita-Salute San Raffaele University, 20122 Milan, Italy; 7 Department of Biomedical, Surgical and Dental Sciences, University of Milan, 20122 Milan, Italy

**Keywords:** liquiritigenin, zebrafish, scale, type 2 diabetes mellitus, osteoporosis, bone

## Abstract

Diabetes mellitus is a metabolic disease characterized by chronic hyperglycemia that induces other pathologies including diabetic retinopathy and bone disease. Adult *Danio rerio* (zebrafish) represents a powerful model to study both glucose and bone metabolism. Then, the aim of this study was to evaluate the effects of liquiritigenin (LTG) on blood glucose level and diabetes complications in hyperglycemic adult zebrafish. LTG is a flavonoid extracted from *Glycyrrhiza glabra* roots which possess important antioxidant, anti-inflammatory, and anti-diabetic properties. During four weeks of glucose treatment, LTG significantly prevented the onset of the hyperglycemia in adult zebrafish. Moreover, hyperglycemic fish showed increased advanced glycation end-products (AGEs) and parathormone levels whereas LTG completely prevented both of these metabolic alterations. Large bone-loss areas were found in the scales of glucose-treated fish whereas only small resorption lacunae were detected after glucose/LTG treatment. Biochemical and histological tartrate resistant acid phosphatase (TRAP) assays performed on explanted scales confirmed that LTG prevented the increase of osteoclastic activity in hyperglycemic fish. The osteoblastic alkaline phosphatase (ALP) activity was clearly lost in scales of glucose-treated fish whereas the co-treatment with LTG completely prevented such alteration. Gene expression analysis showed that LTG prevents the alteration in crucial bone regulatory genes. Our study confirmed that LTG is a very promising natural therapeutic approach for blood glucose lowering and to contrast the development of bone complications correlated to chronic hyperglycemia.

## 1. Introduction

Diabetes mellitus is a metabolic disease characterized by chronic hyperglycemia and impaired insulin secretion or action. Diabetes is one of the major pathological problem of our society because it is associated with highly severe long-term health complications. In fact, chronic hyperglycemia affects a large variety of organs inducing several secondary pathologies including retinopathy and bone alterations [1].

Liquiritigenin (LTG) is a licorice-derived flavonoid (from *Glycyrrhiza glabra*) that is already tested in in vitro and in vivo models for treating many diseases, including diabetes [2,3]. LTG, known for its anti-inflammatory, anti-hyperlipidemic, and antioxidative properties, has already been recognized as an optimum treatment to attenuate diabetic complications derived from hyperglycemia. It has been demonstrated in vitro that LTG protects glomerular mesangial cells (HBZY-1) from high glucose-induced extracellular matrix accumulation, oxidative stress, and the inflammatory response by reducing the production of interleukin 1β (IL1β), interleukin 6 (IL-6), and activation of nuclear factor-kappa B (NF-kB) and nod-like receptor protein 3 (NLRP3) [4]. In vivo studies confirmed its anti-diabetic activity; for example, studies performed on streptozocin-induced diabetic mice have shown that LTG and isoliquiritigenin have a glucose-lowering effect measured through oral glucose tolerance test and also induce a reduction of triglycerides [2,5].

The mechanisms by which type 2 diabetes mellitus (T2DM) induces bone alterations are still debated and not completely clarified but it is well known that hyperglycemia affects bone health in many ways inducing fragility, mechanical strength reduction, impaired bone matrix microstructure, and altered bone cell function, leading to osteoporosis and increased risk of fractures [6,7]. In fact, hyperglycemia leads to hypocalcemia by increasing urinary calcium excretion, impairing the vitamin D status, and interfering with the parathyroid hormone (PTH) and vitamin D axis, moreover, it induces a chronical inflammatory state [8]. High blood glucose levels increase the formation of advanced glycation end-products (AGEs) that are a source of reactive oxygen species (ROS) and that directly damage bone extracellular matrix by creating irreversible cross-links between the collagen type 1 fibers [9]. Moreover, some of the antidiabetic agents, such as thiazolidinediones, can negatively affect bone metabolism through alterations of the transcriptional regulation [6].

LTG is a very promising anti-diabetes therapeutic agent not only for its glucose-lowering activities but also because of its effects on bone metabolism. It has been reported that LTG has beneficial effects on osteoblast activity and bone mineralization process in several in vitro studies. In fact, LTG increases osteoblasts growth, enhances alkaline phosphatase (ALP) activity, collagen synthesis, and significantly reduces ROS production and osteoclasts differentiation through the modulation of tumor necrosis factor α (TNFα), IL-6, and receptor activator of nuclear-kB ligand (RANKL) [10]. Furthermore, our recent study, performed in adult zebrafish glucocorticoid-induced osteoporosis (GIOP) model, highlights the LTG effects in counteracting the osteoporotic complication related to glucocorticoid treatment (Carnovali M, Banfi G, Mariotti M.; unpublished material).

Then, the aim of this study was to test LTG as a possible natural therapeutic and preventive agent for diabetic complications in zebrafish T2DM model focusing on diabetes-induced bone disease.

## 2. Materials and Methods

### 2.1. Ethic Statement

This experimentation has been performed in the Zebrafish Laboratory (IRCCS Galeazzi/GSD Foundation, Milan, Italy) according to the Italian and European guidelines on research (EU Directive 2010/63/EU). Zebrafish experimentation and all protocols of this study were approved by the Italian Ministry of Health (authorization No.349/2017-PR).

### 2.2. Animals

Adult *Danio rerio* of AB strain were maintained under standard conditions [11] in a ZEBTEC© bench top system (Tecniplast, Buguggiate, Italy). The treatments have been performed maintaining fish in E3 medium (5 mM NaCl, 0.17 mM KCl, 0.33 mM CaCl_2_, 0.33 mM MgSO_4_) solution at 28 °C.

### 2.3. Chemicals

Both D-(+)-glucose and liquiritigenin (7,4′-Dihydroxyflavanone, LTG) have been purchased by Sigma-Aldrich. Glucose have been dissolved in E3 medium while LTG was initially dissolved in dimethyl sulfoxide (DMSO, Sigma-Aldrich, St. Louis, Missouri, USA) then diluted in E3 medium, with or without glucose, to have the final concentration of 1 nM.

### 2.4. Adult Treatments and Samples Collection

Zebrafish diabetic model has been generated through immersion in E3 medium 4% glucose solution following our previously published protocol [12] while non-diabetic fish has been maintained in E3 medium only. In order to study LTG effects, both glucose and non-glucose-treated fish have been treated with 1 nM LTG for all the 4 weeks of experimentation. This LTG concentration has been identify in another study (Carnovali M, Banfi G, Mariotti M.; unpublished material) since it has shown biological activity useful to counter the effects of glucocorticoid-induced osteoporosis.

At the end of the treatment, samples have been collected as described in our previously published paper [12]. Briefly, fish were euthanized and blood collection was performed according to a previously published protocol [13]. Basal glycemia measurement was performed according to previously published protocols [13,14] using a glucometer (Freestyle Lite, Abbott, Alameda, CA, USA), the remaining collected blood was pooled and used to quantify advanced glycosylation end products (AGEs) and parathormone (PTH) through ELISA tests (Fish Hemoglobin-Advanced glycosylation End Products ELISA Kit and Fish Parathormone Intact ELISA Kit, MyBioSource, Santiago, CA, USA) according to manufacturer’s protocols. Scales have been carefully removed from both sides of the fish body operating using Dumont^®^ Stainless steel forceps (Sigma Aldrich) under a light stereomicroscope (Olympus SZX-ZB7) and have been processed differently depending on the test to be performed, as described below.

### 2.5. Bone Matrix Vital Staining

At the end of the treatment, fish were live stained overnight using a 0.005% calcein (Bis[*N*,*N*-bis(carboxymethyl)aminomethyl]fluorescein, Sigma Aldrich) E3 solution [12]. Then, after removal as previously described, scales were fixed using 3.5% formaldehyde 0.1 M sodium phosphate buffer solution. Images were acquired and analyzed using a fluorescence microscope (Olympus SZX-ZB7) equipped with Discovery CH30 camera (TiEsseLab, Milano, Italy).

### 2.6. Histological TRAP and ALP Scale Assays

Histological tartrate resistant acid phosphatase (TRAP) assay were performed directly on explanted scales using Leukocytes acid phosphatase (TRAP) detection kit (Sigma Aldrich) following the manufacturer’s protocol. Histological ALP assay was performed using BCIP^®^/NBT liquid substrate (Sigma Aldrich) according to manufacturer’s protocol.

### 2.7. Biochemical TRAP and ALP Scale Assays

Biochemical TRAP and ALP activities were evaluated on explanted scales following previously published methods [15,16]. To perform both these analyses, absorbance was read at 405 nm using a spectrophotometer (iMarkTM Microplate Reader, Bio-Rad, Hercules, CA, USA).

### 2.8. Gene Expression Analysis

Euroclone GOLD total RNA kit (Euroclone, Milano, Italy) was used to isolate total RNA of the explanted zebrafish scales, then Euroscript M-MLV reverse Transcriptase kit (Euroclone, Milano, Italy) was used to perform the complementary DNA (cDNA) synthesis. cDNA was used to perform quantitative real-time PCR (qPCR) to analyze bone metabolism marker genes such as tumor necrosis factor receptor superfamily member 11b (*tnfrsf11b*, fish homolog of human *osteoprotegerin*), tumor necrosis factor (ligand) superfamily member 1B (*tnfrsf1b*, fish homolog of human *rank*), and tumor necrosis factor (ligand) superfamily member 11 (*tnfsf11,* fish homolog of human *rankl*). qPCR was performed using StepOne^TM^ Real-time PCR system (Applied Byosystems, Foster City, CA, USA) using Fluocycle II^TM^ SYBR^®^ Master Mix (Euroclone, Milano, Italy). Gene primers and the amplification conditions were previously published [17]. Each qPCR analysis was run in triplicate. The values of target messenger RNA (mRNA) expression were calculated from the target threshold cycle values and βactin mRNA level was used as standard curve.

### 2.9. Statistics

Three fish were tested for each treatment (control, 4% glucose, LTG and 4% glucose plus LTG) and the entire experiment was repeated three times with comparable results. Data concerning basal glycemia at the 28th day of treatment refers to the mean of six glycemia fish; the remaining blood was used to perform ELISA tests. Data concerning scale biochemical analysis of ALP and TRAP activity were derived from 20 scales for each tested fish, while histological analysis refers to 50 scales for fish. qPCR analysis was repeated three times with comparable results and each qPCR analysis was run in triplicate. Data are expressed as mean ± standard deviation. Statistical significance per *p*-values were set using one-way analysis of variance (ANOVA) followed by Bonferroni test. All significance values were set at less than *p* < 0.05 (*), *p* < 0.01 (**) and *p* < 0.001 (***).

## 3. Results

### 3.1. Liquiritigenin Decreases Blood Alterations in Hyperglycemic Zebrafish Model

**Figure 1:**
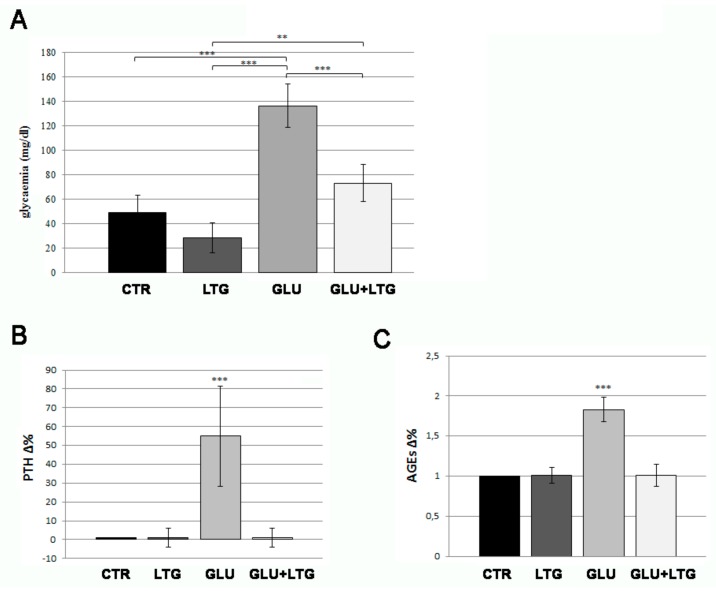
(**A**) Basal blood glucose measurement. Fish treated with glucose (GLU) were compared to control fish (CTR), LTG-treated fish (LTG), and glucose/LTG (GLU + LTG) co-treated fish (CTR vs. GLU *p* < 0.001, LTG vs. GLU *p* < 0.001, GLU + LTG vs. GLU *p* < 0.001). Parathormone (PTH) (**B**) and advanced glycation end-products (AGEs) (**C**) dosage in fish blood. Blood level of AGEs and PTH of glucose-treated fish (GLU) were compared to control (CTR) and glucose/LTG-treated (GLU + LTG) fish (CTR vs. GLU, *p* < 0.001; GLU + LTG vs. GLU, *p* < 0.001). *p* < 0.01 (**) and *p* < 0.001 (***).

Adult fish were treated with 4% glucose solution for four weeks. Glucose treatment induced basal hyperglycemia, whose average results were equal to 137 mg/dL after 28 days of treatment (Figure 1A, +175% vs. CTR). Further, 1 nM LTG treatment partially prevented the onset of the hyperglycemia, reducing basal glycemia to 73, only 48% higher that CTR levels. Moreover, diabetic fish showed increased PTH (Figure 1B) and AGEs (Figure 1C) levels and LTG completely prevented both of these alterations, maintaining levels equal to normal ones.

### 3.2. Liquiritigenin Treatment Reduces Bone Loss in Glucose-Treated Fish

**Figure 2:**
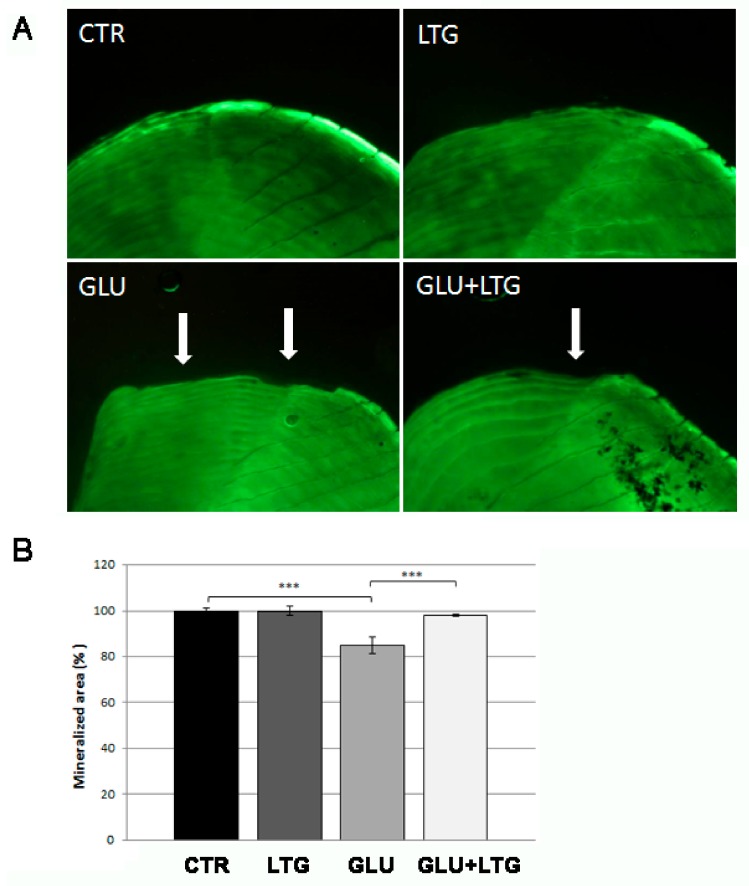
(**A**) Calcein vital staining of fish scales. The mineralized tissue profile can be observed in untreated (CTR) scales or treated with LTG, glucose, and GLU + LTG. Mineralized matrix resorption lacunae are indicated by white arrows. (**B**) Quantification of mineralized area of the scale (CTR vs. GLU, *p* < 0.001; GLU + LTG vs. GLU, *p* < 0.001). *p* < 0.001 (***).

In order to evaluate liquiritigenin effects on hyperglycemia-induced bone loss, we performed calcein vital staining on explanted scales. Great resorption lacunae (Figure 2A, white arrows) were evident on glucose-treated scale border whereas only small resorption lacunae were detected on glucose/LTG co-treated scales. Moreover, we quantified the mineralized area of the scales using imaging software tools, evidencing that the glucose-dependent reduction of 15.2% was significantly prevented by LTG treatment (Figure 2B). LTG alone did not show any effect on scale bone matrix.

### 3.3. Liquiritigenin Prevents Osteoclast Activation in Glucose-Treated Fish

**Figure 3:**
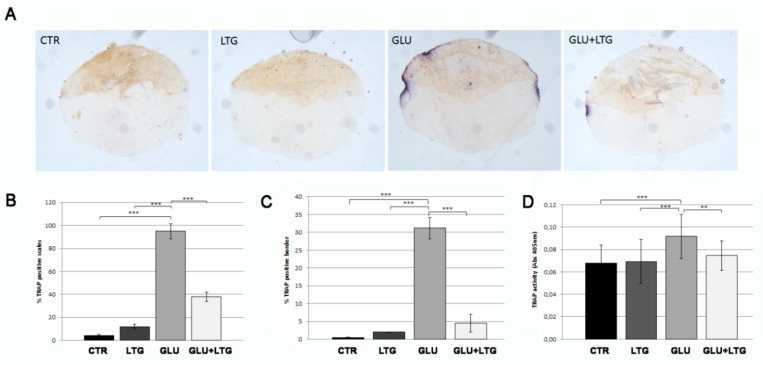
(**A**) Histological TRAP activity assay. Enzymatic activity along scale border of CTR scales or treated with LTG, GLU, and GLU + LTG can be visualized by violet staining. (**B**) Percentage of TRAP-positive scales (CTR vs. GLU, *p* < 0.001) (GLU + LTG vs. GLU, *p* < 0.001). (**C**) TRAP-activity extension calculation among TRAP-positive scales, (CTR vs. GLU, *p* < 0.001) (GLU + LTG vs. GLU, *p* < 0.001). (**D**) Biochemical TRAP assay. Quantification of TRAP activity was performed on same samples with a biochemical assay (CTR vs. GLU, *p* < 0.001; GLU + LTG vs. GLU, *p* < 0.001). *p* < 0.01 (**) and *p* < 0.001 (***).

In order to evaluate the catabolic osteoclast activity, histological and biochemical TRAP assays were performed directly on the explanted scales. Histological TRAP staining evidenced an intense signal along the scale border of glucose-treated fish scales and a significantly reduced TRAP activity on scales from fish treated with both glucose and LTG (Figure 3A). In fact, LTG treatment reduced the percentage of TRAP-positive scales (from 95% to 38%, Figure 3B) and the size of resorption lacunae, measured as percentage of resorbed scale circumference (from 31% to 4.5%, Figure 3C), with respect to glucose-treated fish. Biochemical TRAP assay performed on explanted scales confirmed that LTG treatment prevented the increase of TRAP activity in hyperglycemic fish (Figure 3D). LTG alone did not perturb normal osteoclastic activity.

### 3.4. Liquiritigenin Treatment Prevents the Reduction of Alkaline Phosphatase Activity in Hyperglycemic Fish

**Figure 4:**
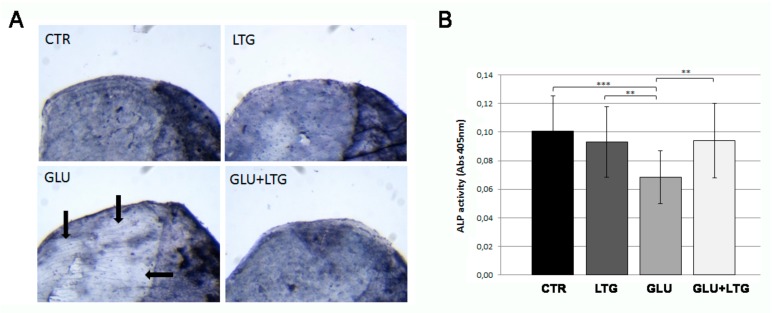
(**A**) Histological ALP activity assay. Enzymatic ALP activity of CTR scales or treated with LTG, GLU, and GLU + LTG can be visualized as blue staining. The areas of fish scale with a reduced ALP activity are evidenced by black arrows. (**B**) Biochemical ALP assay. Quantification of ALP activity was performed on same samples with a biochemical assay (CTR vs. GLU, *p* < 0.001) (GLU + LTG vs. GLU, *p* < 0.01). *p* < 0.01 (**) and *p* < 0.001 (***).

In order to evaluate the anabolic osteoblast activity, histological and biochemical ALP assays were performed directly on the explanted scales. In the histological assay, the ALP signal was clearly lost in scales from glucose-treated fish whereas the co-treatment with LTG was able to completely prevent such alterations (Figure 4A). These data were confirmed by the biochemical analysis that showed a reduction of 32% ALP activity in glucose-treated scale, whereas in the presence of LTG, the reduction is limited to 6% (Figure 4B).

### 3.5. Liquiritigenin Treatment Prevents the Alteration of Bone Regulatory Genes in Hyperglycemic Fish

**Figure 5:**
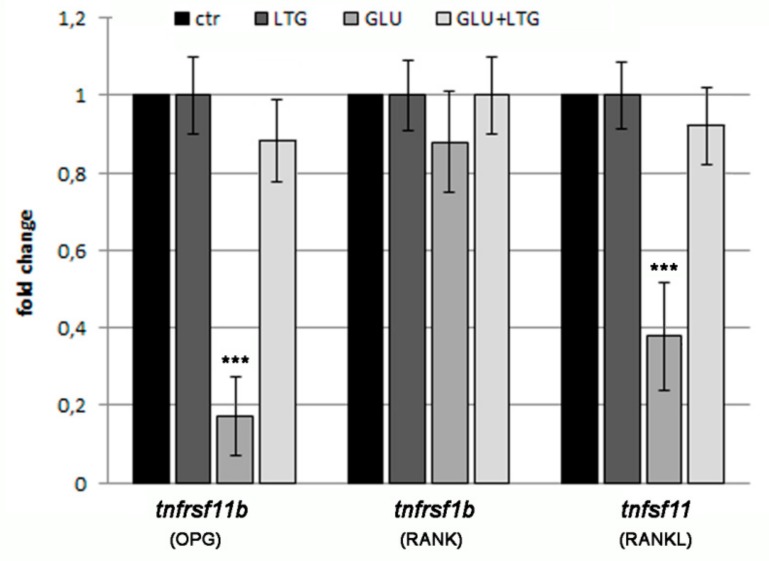
qPCR analysis of bone marker genes. Bone markers related to bone metabolism have been analyzed: Tumor necrosis factor receptor superfamily member 11b (*tnfrsf11b*, homolog of osteoprotegerin OPG), tumor necrosis factor receptor superfamily member 1B (*tnfrsf1b*, homolog of RANK), and tumor necrosis factor (ligand) superfamily member 11 (*tnfsf11*, homolog of RANKL). (CTR vs. GLU, *p* < 0.001). *p* < 0.001 (***).

The expression analysis of genes involved in the regulation of bone tissue was done by real-time PCR in the scales after treatment with glucose and/or LTG (Figure 5). Tumor necrosis factor receptor superfamily, member 11b *tnfrsf11b* (fish homolog of human *osteoprotegerin*), and tumor necrosis factor (ligand) superfamily member 11 *tnfrsf11* (fish homolog of human *rankl*), two important regulators of osteoclast differentiation, were found decreased in the scales of hyperglycemic fish, whereas tumor necrosis factor receptor superfamily member 1B *tnfrsf1b* (fish homolog of human *rank*) resulted in being not modulated. LTG co-treatment restored, in the GLU-treated fish, the expression level found in CTR and LTG alone.

## 4. Discussion

The anti-inflammatory effect of LTG has been widely documented through several studies that highlights its role in the modulation of pro-inflammatory cytokines such as IL-1β and IL-6 [18]. Because of very promising in vitro and in vivo studies in diabetic-related bone disease, LTG can be considered a potential candidate to develop a new therapeutic approach against diabetes-related effects [19,20]. LTG tested in our T2DM zebrafish model confirms its ability to contrast the onset of basal glycemia after glucose treatment. In fact, a similar effect has been previously reported in vivo in streptozocin-nicotinamide diabetic mice during oral glucose tolerance test [2,5]. As expected in a diabetes animal model, we detected a significant increase of AGEs in the blood of hyperglycemic fish by ELISA and co-treatment with LTG was able to suppress AGEs generation suggesting its potential ability to reduce hyperglycemia-induced complications.

The chronic hyperglycemia in diabetic patients promotes AGEs generation that, in turn, induces cellular dysfunction and other tissue damages by acting on their receptor RAGE (receptor for AGE) [21].

About diabetes complications, glycemia and AGEs have been positively associated with bone turnover in older men, with increasing incidence of hip fracture [22,23].

We can hypothesize that the increase of AGEs can contribute to bone loss induction in our model of hyperglycemic zebrafish. Several alternative therapeutic approaches against AGEs in diabetes patients are currently being studied, including the use of natural molecules [24]. The capacity of LTG to contrast the generation of AGEs and bone loss phenotype is pivotal to further understand if this molecule is able to prevent other diabetes-related complications such as retinopathy, nephropathy, and neuropathy.

We also detected a significant increase of PTH level in the blood of hyperglycemic fish by ELISA assay. The hormonal regulation of calcium metabolism is crucial in human bones and the PTH pathway plays an important role [25]. It has been demonstrated that high levels of PTH can directly affect osteoclast activity leading to an increased bone resorption [26]. There are conflicting data concerning PTH role in the glucose homeostasis but most studies agree that impaired glucose metabolism is associated with elevated PTH levels and that there is a correlation between PTH and insulin resistance [27]. These data support the idea that an increase of PTH levels in hyperglycemic fish can represent a positive stimulus for osteoclast activation. The effect of LTG in lowering PTH level may represent additional evidence of the anti-osteoporotic activity of LTG. In fact, in our zebrafish model, we correlated the anti-diabetic properties of LTG to the ability to maintain the normal bone metabolism in fish scales. As previously described [12], hyperglycemia is correlated to a highly significant increase of osteoclast activity and decrease of osteoblast activity that led to an osteoporotic phenotype in adult fish scales. LTG has already been reported to have positive effects on osteoblasts activity and on the reduction of osteoclast differentiation in vitro [10]. Studies performed on MC3T3-E1 cells showed that LTG promotes their osteoblast differentiation dose-dependently acting on the Smad1/5-dependet pathway, increases ALP activity, collage synthesis, and their mineralization. [28,29].

A study performed on RANKL-stimulated RAW-D cells induced to differentiate osteoclasts highlights that LTG inhibits both the formation of mononuclear and multinuclear osteoclasts and impairs the bone resorption activity of BMM-derived osteoclasts. Moreover, LTG has been reported to decrease osteoclasts differentiation factors such as TNFα, IL-6, and RANKL [10].

Moreover, we recently reported important effects of LTG in counteracting the bone-loss phenotype in zebrafish GIOP model (Carnovali M, Banfi G, Mariotti M.; unpublished material). LTG treatment in T2DM model can sensitively reduce the onset of the osteoporosis phenotype, contrasting the osteoclast activation and protecting osteoblast activity. It is known that high-glucose microenvironment can stimulate an inflammatory status with increased production of cytokines, AGEs, and ROS, which are able to induce osteoclastogenesis [30]. Recent studies on implant cores evidenced that bone formation activities by osteoblasts were suppressed in the presence of elevated AGEs, which ultimately results in the development of bone loss [31].

Regarding the molecular mechanism of LTG protective effects on hyperglycemic-induced bone loss, the gene expression analysis suggested that LTG suppresses the reduction of osteoclastogenesis-inhibitor factor *tnfrsf11b* (osteoprotegerin) and *tnfsf11* (rankl). Further studies will be involved in the analysis of inflammatory and oxidative stress pathways in hyperglycemic fish and the role of LTG as therapeutic modulator.

## 5. Conclusions

LTG treatment in hyperglycemic fish is able to improve blood glucose clearance preventing the generation of AGEs and the alteration of calcium metabolism (PTH pathway). In this way, bone turnover and resorption cannot be activated, maintaining the normal equilibrium of bone metabolism in the scales.

Our study confirmed that LTG is a very promising natural therapeutic approach for blood glucose lowering and to contrast the development of bone complications correlated with chronic hyperglycemia. Further studies will evaluate the effect of LTG in preventing other diabetes-related complications such as retinopathy, nephropathy, and neuropathy.
